# Neurologic Symptoms in Licensed Private Pesticide Applicators in the Agricultural Health Study

**DOI:** 10.1289/ehp.7645

**Published:** 2005-04-15

**Authors:** Freya Kamel, Lawrence S. Engel, Beth C. Gladen, Jane A. Hoppin, Michael C. R. Alavanja, Dale P. Sandler

**Affiliations:** ^1^National Institute of Environmental Health Sciences, National Institutes of Health, Department of Health and Human Services, Research Triangle Park, NC, USA; ^2^Memorial Sloan-Kettering Cancer Center, New York, New York, USA; ^3^National Cancer Institute, National Institutes of Health, Department of Health and Human Services, Rockville, Maryland, USA

**Keywords:** fumigants, insecticides, neurologic symptoms, organochlorines, organophosphates, pesticide applicators, pesticides

## Abstract

Exposure to high levels of many pesticides has both acute and long-term neurologic consequences, but little is known about the neurotoxicity of chronic exposure to moderate levels of pesticides. We analyzed cross-sectional data from 18,782 white male licensed private pesticide applicators enrolled in the Agricultural Health Study in 1993–1997. Applicators provided information on lifetime pesticide use and 23 neurologic symptoms typically associated with pesticide intoxication. An indicator of more symptoms (≥10 vs. < 10) during the year before enrollment was associated with cumulative lifetime days of insecticide use: odds ratios (95% confidence intervals) were 1.64 (1.36–1.97) for 1–50 days, 1.89 (1.58–2.25) for 51–500 days, and 2.50 (2.00–3.13) for > 500 days, compared with never users. A modest association for fumigants [> 50 days, 1.50 (1.24–1.81)] and weaker relationships for herbicides [> 500 days, 1.32 (0.99–1.75)] and fungicides [> 50 days, 1.23 (1.00–1.50)] were observed. Pesticide use within the year before enrollment was not associated with symptom count. Only associations with insecticides and fumigants persisted when all four pesticide groups were examined simultaneously. Among chemical classes of insecticides, associations were strongest for organophosphates and organochlorines. Associations with cumulative exposure persisted after excluding individuals who had a history of pesticide poisoning or had experienced an event involving high personal pesticide exposure. These results suggest that self-reported neurologic symptoms are associated with cumulative exposure to moderate levels of fumigants and organophosphate and organochlorine insecticides, regardless of recent exposure or history of poisoning.

Pesticides are used extensively throughout the world. In the United States, > 18,000 products are licensed for use, and annual use of pesticides for crops, homes, schools, parks, and forests exceeds 2 billion pounds [U.S. Environmental Protection Agency (EPA) 2002]. Neurologic dysfunction is the best-documented health effect of pesticide exposure. High-level exposure has both acute and long-term neurologic effects, and adverse effects have been reported for most types of pesticides, including organophosphate, carbamate, organochlorine, and pyrethroid insecticides, herbicides, fungicides, and fumigants. Organophosphates have been studied in greatest detail. Acute organophosphate poisoning can involve a wide range of both central and peripheral neurologic symptoms (Ecobichon 1996; Keifer and Mahurin 1997). Effects of organophosphate poisoning may persist long after the acute response is resolved; sequelae include increased neurologic symptoms, deficits in neurobehavioral performance, decreased vibration sensitivity, and impaired nerve conduction (Kamel and Hoppin 2004). Effects continue up to 10 years after poisoning (Savage et al. 1988), suggesting permanent residual damage. Even less severe poisoning can have long-term consequences (Wesseling et al. 2002).

Questions remain concerning the neurologic effects of moderate pesticide exposure. Most studies show effects on cognitive and psychomotor neurobehavioral function in chronically exposed individuals without a history of poisoning, although clinical measures of peripheral nerve function like vibration sensitivity and nerve conduction are not generally affected (Kamel and Hoppin 2004). Increases in both central and peripheral neurologic symptoms are also found in many studies of moderate exposure (Kamel and Hoppin 2004). Increased symptom prevalence may provide early evidence of neurologic dysfunction, before clinically measurable signs are evident. Unresolved issues regarding the relationship of pesticide exposure to symptom prevalence include the relative importance of acute and chronic exposure, of pesticide poisoning or high-exposure events compared with chronic moderate exposure, and of pesticides other than organophosphates.

The Agricultural Health Study (AHS) is a large cohort study of licensed pesticide applicators and their spouses (Alavanja et al. 1996, 1999a). Questionnaires completed by applicators at enrollment provided information on neurologic symptoms during the prior year as well as detailed information on lifetime pesticide use and exposure. We used this information for a cross-sectional analysis of the relationship of symptoms to several measures of pesticide exposure.

## Materials and Methods

### Population and questionnaires.

The AHS cohort was recruited in 1993–1997 (Alavanja et al. 1996, 1999a). In Iowa and North Carolina, licenses for restricted-use pesticides must be renewed every 3 years. Individuals applying for new or renewed licenses were invited to enroll in the study. Approximately 52,400 private applicators (mostly farmers) participated, 82% of those eligible. At enrollment, participants completed a self-administered questionnaire that collected information on demographic characteristics, lifestyle, medical history, and pesticide use. A supplemental questionnaire, completed at home by 44% of the enrolled private applicators, collected additional information in these categories as well as information on neurologic symptoms. Applicators who returned the supplemental questionnaire were similar to those who did not in most respects, including prevalence of symptoms and pesticide exposure (Tarone et al. 1997). Together the two questionnaires collected information on frequency and duration of use of 50 specific pesticides as well as on high-pesticide-exposure events, medical visits for pesticide-related illness, and pesticide poisoning. All information on exposures and disease states was taken from these self-reports. Symptom questions were based on an established questionnaire, Q16, previously used to evaluate effects of occupational exposure to neurotoxicants (Lundberg et al. 1997). The questionnaires are available on the AHS website (AHS 2004); the symptom questions are in section 8, Medical History, in the Farmer Applicator Questionnaire.

The present analysis was restricted to applicators with complete information on symptoms—89% of those who returned the supplemental questionnaire. Applicators with incomplete symptom information (*n* = 2,603) were slightly older, less well educated, and more likely to live in North Carolina, compared with the analysis group, but the median number of symptoms experienced at least once was the same. Many of the excluded applicators omitted information for only one or two symptoms (*n* = 1,651, 63% of those excluded). Including them in the analysis by imputing either a positive or negative response for the missing symptoms did not substantively change results. To reduce heterogeneity, the analysis was further restricted to white men 18–75 years of age, who comprised 92% of those otherwise eligible. Characteristics of the 18,782 applicators included in the analysis are presented in Table 1.

The institutional review boards of the National Institutes of Health, the University of Iowa (Iowa field station), and Battelle (North Carolina field station) approved the AHS. The study was explained to potential participants, who indicated consent by returning questionnaires.

### Data analysis.

Table 2 shows frequencies of 23 symptoms during the year before enrollment, in the categories originally reported. Most of our analyses focused on these symptoms as a group. We created two measures reflecting the number of symptoms experienced at least once in the year before enrollment: a continuous variable, “number of symptoms” [median (interquartile range) = 4 (1–8)], and a dichotomous variable, “many symptoms,” that compared the 20% of applicators who experienced ≥10 symptoms (“cases”) with the remaining 80% who experienced < 10 symptoms (“controls”). The distribution of number of symptoms was not greatly affected by omitting the two most common and nonspecific symptoms, headache and fatigue. We also considered the 23 symptoms individually, dichotomizing the frequency of each symptom so that 5–15% of the population was in the positive (“case”) category (Table 2).

We considered several pesticide exposure measures, listed in Table 3. Applicators reported years of use of any pesticide (in five categories) and days per year (in seven categories). We then calculated lifetime days of use by multiplying duration (quantified as the central value of the reported category for years of use) by frequency (central value of the reported category for days per year) and then categorizing in quartiles. A high-exposure event was one causing “unusually high personal exposure” (Alavanja et al. 1999b). Internal exposure was defined as inhalation or ingestion of pesticide. Pesticides were categorized by function or mode of use, as herbicides, insecticides, fungicides, or fumigants (Table 3). Insecticides were further categorized by chemical class. Cumulative lifetime days for pesticide groups were calculated by multiplying duration by frequency of use for each pesticide in the group, summing over all pesticides in the group, and categorizing into three or four levels. We further categorized by whether or not any pesticides in the group were used in the year before enrollment (Table 3).

We analyzed the data using linear regression for number of symptoms and logistic regression for the dichotomous outcomes. Analyses were performed in SAS (version 8.2; SAS Institute Inc., Cary, NC) using AHS phase I data (Prerelease 9/02; AHS 2004). We adjusted for age, state, education, smoking, and alcohol use, with variables categorized as in Table 1. Information on age and state was available for all applicators. For applicators (< 3%) who were missing data on education, cigarette smoking, or alcohol use, we imputed the median value for their state and age category. All outcome measures had similar relationships to covariates. Final models included two-way interactions for age × state, age × education, age × smoking, age × drinking, state × education, state × smoking, and state × drinking.

## Results

In multivariate analyses, symptom prevalence was greater among applicators who were from Iowa, had more than a high school education, smoked more, and drank more. There was a strong monotonic inverse association of symptoms with age: Applicators 66–75 years of age had 2.2 fewer symptoms and were half as likely to have ≥10 symptoms than did applicators 18–30 years of age.

Both measures of symptom count—having many (≥10) symptoms and total number of symptoms—were associated with overall pesticide use (Table 3). Applicators in the highest quartile of lifetime days of pesticide use were 1.2 times as likely to experience ≥10 symptoms and averaged 0.6 more symptoms in the year preceding enrollment, compared with the lowest quartile, with a weak dose–response relation across quartiles. Individuals with a history of pesticide poisoning experienced more symptoms than did those without such a history (Table 3). Applicators who had ever sought medical attention for pesticide-related illness also experienced more symptoms than those who had not (Table 3), even when individuals with a history of pesticide poisoning were excluded (doctor visit: 2.1 times more likely to have ≥10 symptoms and 1.8 more symptoms). Experiencing a high-exposure event was associated with an increased symptom count, particularly when internal exposure (inhalation or ingestion of pesticide) was involved (Table 3). Again, similar results were seen when individuals with a history of pesticide poisoning were excluded (risk estimates for internal exposure were unchanged).

Pesticides were grouped in functional categories, and cumulative use (lifetime days) and recent use (in the year before enrollment) were evaluated simultaneously. Cumulative use of insecticides was associated with both measures of symptom count, with a pronounced dose response (Table 3). Weaker associations of symptoms with cumulative herbicide, fungicide, and fumigant use were observed. After accounting for cumulative use, recent use was not associated with an increased symptom count for any pesticide category (Table 3). Associations with cumulative use were similar in models not including recent use (data not shown). When cumulative use in all four functional categories was assessed simultaneously, associations with insecticides and fumigants were only slightly reduced, but relationships with herbicides and fungicides were no longer present; results were similar whether or not recent use was included in the models (data not shown).

Insecticides were further categorized by chemical class, with cumulative and recent use evaluated simultaneously. Greater symptom count was associated with cumulative use in all four chemical classes of insecticides (Table 3). Associations were strongest for organophosphates and organochlorines; dose response for cumulative use was evident for all classes except pyrethroids. After accounting for cumulative use, recent use was not associated with symptom count for any chemical class (Table 3). Associations with cumulative use were similar in models not including recent use (data not shown). When cumulative use in all four chemical classes was considered simultaneously, associations with organophosphates, organochlorines, and carbamates were still present, although slightly attenuated, but the relationship with pyrethroids was present only for number of symptoms and not for many symptoms; Results were similar whether or not recent use was included in the models (data not shown).

Associations with cumulative use of pesticides in functional or chemical groups were not affected by excluding individuals with diagnosed pesticide poisoning (*n* = 363) or those who had experienced a high-exposure event (*n* = 2,688). In models including cumulative use of all four functional categories of pesticides, applicators in the highest category of insecticide use were 2.2–2.4 times as likely to experience ≥10 symptoms and averaged 2.0–2.1 additional symptoms, regardless of whether or not individuals with pesticide poisoning, a high-exposure event, or either were excluded from the analysis. In models including cumulative use in all four chemical classes of insecticides, applicators in the highest category of organophosphate use were 1.6–1.7 times as likely to experience ≥10 symptoms and averaged 1.0–1.1 additional symptoms, regardless of exclusions. Associations of symptoms with cumulative pesticide use were also not affected by excluding individuals with self-reported neurologic disease (*n* = 498), depression (*n* = 849), stroke (*n* = 113), head injury (*n* = 2,307), myocardial infarction (*n* = 518), or diabetes (*n* = 635); these were similar in Iowa and North Carolina and were not affected by adjusting for occupational exposure to solvents or metals on or off the farm (data not shown).

Specific symptoms were also related to pesticide use. Symptoms were associated with use of any pesticide, any insecticide, organophosphates, organochlorines, or fumigants (Table 4). Pesticide poisoning, pesticide-related medical visits, and high-exposure events were also associated with increases in specific symptoms but did not alter the observed associations with pesticide use (data not shown). For any particular exposure measure, there was little variation in the magnitude of the associations among symptoms. Similar results were found when symptoms were grouped in categories defined *a priori* to reflect particular aspects of neurologic function, including affect, cognition, autonomic function, motor function, and vision (data not shown).

## Discussion

In this study we found that increased symptom count was associated with cumulative lifetime use of pesticides, particularly insecticides and fumigants. Increased symptom count was also associated with a history of pesticide poisoning or events involving high personal pesticide exposure. Significantly, however, associations with cumulative use persisted even after excluding individuals with a history of pesticide poisoning or high exposure events. Recent pesticide use, within the year before reporting symptoms, was not related to symptom count after accounting for cumulative exposure, and adjustment for recent use did not affect the association of cumulative use with symptom count.

Most previous studies of pesticides and neurologic symptoms have focused on organophosphates. Farm workers (Gomes et al. 1998; Strong et al. 2004), greenhouse workers (Bazylewicz-Walczak et al. 1999), and factory workers (Bellin and Chow 1974) exposed to organophosphates reported more symptoms than unexposed workers. Farmers and farm workers who applied organophosphates had higher symptom prevalence than did nonapplicators (London et al. 1998; Ohayo-Mitoko et al. 2000; Smit et al. 2003), as did commercial termiticide applicators (Steenland et al. 2000) and sheep dippers (Pilkington et al. 2001). Other studies have used the Profile of Mood States or other scales to evaluate changes in mood, finding higher levels of tension, anxiety, anger, and depression in workers exposed to organophosphates (Bazylewicz-Walczak et al. 1999; Levin et al. 1976; Steenland et al. 2000; Stokes et al. 1995). Most (Bellin and Chow 1974; Gomes et al. 1998; Leng and Lewalter 1999; Ohayo-Mitoko et al. 2000) although not all (Ciesielski et al. 1994; Lee et al. 2003) studies found increased symptom prevalence associated with inhibition of erythrocyte acetylcholinesterase activity, a bio-marker of recent organophosphate exposure.

Although poisoning by high exposures to organochlorines, fungicides, and fumigants as well as organophosphates is well documented, and carbamates, pyrethroids, and herbicides are also neurotoxic (Ecobichon 1996; Keifer and Mahurin 1997), questions remain concerning the effects of moderate exposure to pesticides other than organophosphates. One study of moderate exposure found that dichlorodiphenyltrichoroethane (DDT) was associated with increased symptom prevalence (van Wendel de Joode et al. 2001), as did one study of fumigants (Anger et al. 1986), but not another (Calvert et al. 1998). We found that symptom count was related to all classes of pesticides examined, although associations with herbicides and fungicides appeared to be due to confounding by insecticide use. Organophosphate, carbamate, and organochlorine insecticides were independently associated with increased risk. The relative neurotoxicity of specific chemicals or chemical classes may differ for acute high-level and chronic moderate exposure. For example, the stronger effects that we observed for organochlorines may be related to their long biologic half-lives (Ecobichon 1996).

Few previous studies were able to distinguish between effects of acute and chronic exposure because the two are often correlated. Two studies with sufficient information to make the distinction found that in farmworkers who applied pesticides increased symptom prevalence was associated with acute but not chronic exposure (London et al. 1998; Ohayo-Mitoko et al. 2000). In contrast, our results suggest that at moderate levels cumulative lifetime exposure has a greater impact on symptom prevalence than exposure during the year before reporting symptoms. This disparity may be due to the higher level of exposure experienced by farm workers compared with licensed applicators.

The role of pesticide poisoning in the apparent effects of cumulative use is still a question. We confirmed previous reports that a history of pesticide poisoning is associated with increased symptom prevalence (Kamel and Hoppin 2004). A notable finding in our study is that a history of events involving high personal pesticide exposure conferred equally great risk, even in the absence of diagnosed poisoning. Some studies have not differentiated exposed individuals with a history of pesticide poisoning from those without. Two studies that excluded poisoned individuals found no relationship of moderate organophosphate exposure to symptom prevalence (Ames et al. 1995; Fiedler et al. 1997), although a study of DDT that excluded poisoned individuals did find an association (van Wendel de Joode et al. 2001). We found dose-related associations of symptom count to cumulative exposure to all insecticides, organophosphates, and organochlorines whether or not we excluded individuals with a history of pesticide poisoning or those who had experienced high-exposure events, indicating that moderate exposure itself is associated with increased risk.

Our findings were similar regardless of whether we considered summary measures of all symptoms, individual symptoms, or symptom groups defined *a priori*. These results are consistent with previous studies suggesting that moderate pesticide exposure is associated with a wide range of symptoms, reflecting cognitive, sensory, and motor dysfunction and affecting both the central and peripheral nervous systems (Kamel and Hoppin 2004). Pesticide exposure may be associated with some fundamental disorder, such as depression or neurologic disease, which then influences the experience or perhaps the reporting of multiple symptoms. Similarly, confounding by head injury, which was related to pesticide exposure in the AHS cohort, might explain some of the increase in symptoms. However, our findings were not affected by excluding individuals with depression, neurologic disease, or head injury. The earliest manifestation of neurotoxicity after moderate pesticide exposure may in fact be an increase in many symptoms, not restricted to particular aspects of neurologic function. A similar increase in a wide range of symptoms is associated with solvent exposure in mild cases of chronic solvent-related toxic encephalopathy (van der Hoek et al. 2000; White and Proctor 1997).

Confounding by demographic factors does not appear to explain our results. There was a strong inverse association of symptom prevalence with age. The basis of this association is unclear; it may represent participants’ understanding of the symptom questions or reporting proclivities rather than a real relationship. Other explanations are possible. Symptomatic individuals may have left farming at an early age and thus never entered our cohort, representing a type of healthy worker effect. Younger applicators used more pesticides in the year before enrollment, the period for which symptom prevalence was reported; however, adjusting for recent use did not affect associations with cumulative use. In any case, because symptom prevalence decreased and cumulative exposure increased with age, confounding by age or age-related conditions like heart disease or diabetes cannot explain the positive associations we observed with cumulative exposure; moreover, excluding individuals with the latter conditions did not affect our results. This point is particularly important in interpreting results for organochlorine pesticides. Secular trends in use mean that older applicators are more likely to have used these chemicals, but this cannot account for the association with symptoms because age was inversely related to symptom count. We adjusted for other potential confounders, including education, so these are also unlikely to account for our results. We had no information on personality traits that may have affected symptom reporting, and so could not adjust for these, but they are unlikely to have covaried with exposure, particularly in a way that could account for the dose–response relationships we observed.

Potential bias is also a concern. The present analysis was based on the subset of private applicators who completed the take-home questionnaire. Although these are only 44% of the private applicators enrolled in the AHS, they are clearly representative of the cohort as a whole: Applicators who did or did not complete the take-home questionnaire were similar for every lifestyle or demographic characteristic except age; for health outcomes, including experience of pesticide-related health symptoms; and for farm characteristics and tasks and a variety of measures of pesticide exposure (Tarone et al. 1997). These results mitigate concerns regarding selection bias. Because symptoms were self-reported, another concern is potential recall or reporting bias. However, the fact that only some pesticides were associated with symptoms suggests that recall bias does not account for our findings. Risks associated with insecticide exposure were dose-related, further suggesting that bias does not explain our results. Moreover, our findings are biologically plausible, because we found the greatest risk for insecticides, which are designed to be neurotoxicants.

An important strength of our study is its large size. Further, because farming practices are considerably different in Iowa and North Carolina, the AHS cohort represents a diverse farming population (Alavanja et al. 1996, 1999a). We used internal comparisons of more and less exposed individuals from the same population, thereby reducing potential confounding. The primary strength of the study is, however, the availability of detailed exposure information. Although the present analysis is limited by its cross-sectional design, data on symptoms and pesticide exposure were collected in separate portions of the questionnaires, some completed at different times, minimizing potential bias. Exposure data were reported by the applicators themselves, but farmers in general and AHS cohort members in particular report pesticide use reliably (Blair et al. 2002; Hoppin et al. 2002).

In conclusion, we found that prevalence of neurologic symptoms was associated with cumulative lifetime exposure to pesticides, particularly organophosphate and organochlorine insecticides and fumigants. These associations were present in individuals with no history of pesticide poisoning or high exposure events and were independent of recent exposure. Thus, they are likely due to chronic moderate exposure. Although the neurotoxicity of high-level exposure is accepted, more attention to the risks associated with moderate exposure may be required.

## Figures and Tables

**Table 1:** Characteristics of licensed pesticide applicators enrolled in the AHS 1993–1997 (*n* = 18,782).*^a^*

Characteristic	Percent
Age (years)	
18–30	8
31–35	9
36–40	13
41–45	14
46–50	12
51–55	12
56–60	12
61–65	10
66–75	10
State	
Iowa	70
North Carolina	30
Education*^b^*	
≤High school	56
> High school	44
Cigarette smoking (lifetime pack-years)*^b^*	
0	57
> 0–30	35
> 30	8
Alcohol use (any consumption in last year)*^b^*	
No	34
Yes	66

a The analysis was restricted to white men who provided a response for each of 23 neurologic symptoms.

b Missing data (< 3%) were imputed on an age- and state-specific basis for education, cigarette smoking, and alcohol use, using medians of the relevant strata.

**Table 2:** Percentage of study participants experiencing the indicated frequency of neurologic symptoms in the year before enrollment, among licensed pesticide applicators enrolled in the AHS 1993–1997 (*n* = 18,782).*^a^*

Symptom	Never	Once a year	Once a month	Once a week	> Once a week
Headache	32	18	36	10*	4*
Fatigue	42	16	24	10	9*
Tension	48	16	22	9*	6*
Insomnia	57	10	19	8*	7*
Irritability	63	13	16	5*	3*
Dizziness	72	19	7*	1*	1*
Numbness in hands or feet	73	9	10	4*	5*
Depression	73	12	10*	3*	2*
Nausea	73	24	3*	< 1*	< 1*
Absentmindedness	76	7	9	4*	4*
Difficulty concentrating	80	7	7*	3*	2*
Loss of appetite	82	11	5*	1*	< 1*
Excessive sweating	83	8	5*	2*	2*
Twitches in arms or legs	83	7	6*	2*	2*
Fast heart rate	85	7	5*	2*	1*
Weakness in arms or legs	85	6	5*	1*	2*
Poor balance	88	7	3*	1*	1*
Poor night vision	88	3	3*	1*	4*
Tremor in hands	89	5*	3*	1*	2*
Blurred/double vision	90	5*	3*	1*	1*
Changes in smell or taste	94	4*	1*	< 1*	< 1*
Difficulty speaking	96	2*	1*	1*	1*
Loss of consciousness	98	1*	< 1*	< 1*	< 1*

a Symptoms are listed in order of decreasing frequency. Symptom distributions were dichotomized so that the positive category included between 5 and 15% of participants.

*Responses included in the positive (“case”) category.

**Table 3:** Association of summary measures of neurologic symptom prevalence with pesticide use and exposure among licensed pesticide applicators enrolled in the AHS 1993–1997 (*n* = 18,782).

Exposure	Case (%)	Control (%)	Many symptoms*^a^* [OR (95% CI)]	No. symptoms*^b^* [β(SE)]
Use of any pesticide				
Cumulative lifetime days of use				
0–64	23	25	1.00 (referent)	0 (referent)
65–200	22	21	1.16 (1.04–1.30)	0.45 (0.10)
201–396	27	26	1.14 (1.02–1.26)	0.47 (0.10)
397–7,000	28	27	1.20 (1.08–1.33)	0.60 (0.10)
Ever diagnosed with pesticide poisoning				
No	96	98	1.00 (referent)	0 (referent)
Yes	4	2	2.46 (1.97–3.08)	2.58 (0.25)
Ever received medical attention for pesticide-related illness				
No	89	95	1.00 (referent)	0 (referent)
Saw doctor only	9	4	2.25 (1.95–2.59)	2.07 (0.16)
Hospitalized	2	1	1.98 (1.43–2.74)	1.76 (0.35)
Ever had an event involving high personal exposure				
No event	76	88	1.00 (referent)	0 (referent)
Yes, no internal exposure*^c^*	13	8	1.80 (1.60–2.02)	1.68 (0.12)
Yes, with internal exposure*^c^*	11	4	3.04 (2.65–3.49)	3.08 (0.15)
Cumulative pesticide use, functional categories				
Insecticides (lifetime days, without or with use in past year)				
0 days	5	9	1.00 (referent)	0 (referent)
1–50 days, no use in past year	14	15	1.64 (1.36–1.97)	1.25 (0.15)
1–50 days, used in past year	8	10	1.34 (1.09–1.63)	0.90 (0.17)
51–500 days, no use in past year	22	22	1.89 (1.58–2.25)	1.67 (0.14)
51–500 days, used in past year	32	31	1.82 (1.53–2.15)	1.72 (0.14)
> 500 days, no use in past year	6	5	2.50 (2.00–3.13)	2.34 (0.20)
> 500 days, used in past year	12	9	2.50 (2.06–3.03)	2.43 (0.17)
Herbicides (lifetime days, without or with use in past year)				
0 days	2	3	1.00 (referent)	0 (referent)
1–50 days, no use in past year	5	5	1.04 (0.77–1.40)	0.57 (0.27)
1–50 days, used in past year	5	6	0.85 (0.63–1.15)	0.28 (0.26)
51–500 days, no use in past year	13	13	1.16 (0.89–1.52)	0.79 (0.24)
51–500 days, used in past year	36	36	1.11 (0.86–1.43)	0.80 (0.23)
> 500 days, no use in past year	7	7	1.32 (0.99–1.75)	1.10 (0.26)
> 500 days, used in past year	32	30	1.27 (0.98–1.64)	1.25 (0.23)
Fungicides (lifetime days, without or with use in past year)				
0 days	69	71	1.00 (referent)	0 (referent)
1–50 days, no use in past year	12	10	1.31 (1.16–1.48)	0.94 (0.12)
1–50 days, used in past year	9	8	1.21 (1.05–1.39)	0.60 (0.13)
> 50 days, no use in past year	4	4	1.23 (1.00–1.50)	0.60 (0.19)
> 50 days, used in past year	8	7	1.24 (1.05–1.45)	0.67 (0.15)
Fumigants (lifetime days, without or with use in past year)				
0 days	78	81	1.00 (referent)	0 (referent)
1–50 days, no use in past year	13	11	1.48 (1.31–1.66)	1.06 (0.11)
1–50 days, used in past year	3	3	1.16 (0.90–1.48)	0.19 (0.23)
> 50 days, no use in past year	5	4	1.50 (1.24–1.81)	0.99 (0.18)
> 50 days, used in past year	2	2	1.29 (0.98–1.71)	0.56 (0.27)
Cumulative insecticide use, chemical classes				
Organophosphates (lifetime days, without or with use in past year)				
0 days	9	14	1.00 (referent)	0 (referent)
1–50 days, no use in past year	20	21	1.39 (1.20–1.60)	1.00 (0.12)
1–50 days, used in past year	11	12	1.25 (1.06–1.46)	0.80 (0.14)
51–500 days, no use in past year	21	20	1.63 (1.41–1.88)	1.36 (0.12)
51–500 days, used in past year	29	27	1.59 (1.39–1.83)	1.47 (0.12)
> 500 days, no use in past year	4	3	2.16 (1.71–2.73)	1.91 (0.23)
> 500 days, used in past year	6	4	2.23 (1.84–2.71)	2.08 (0.19)
Organochlorines (lifetime days, without or with use in past year)				
0 days	51	56	1.00 (referent)	0 (referent)
1–50 days, no use in past year	29	27	1.60 (1.45–1.75)	1.24 (0.09)
1–50 days, used in past year	1	1	1.04 (0.61–1.74)	0.74 (0.48)
> 50 days, no use in past year	18	16	2.00 (1.78–2.23)	1.76 (0.10)
> 50 days, used in past year	1	1	2.23 (1.51–3.31)	1.74 (0.42)
Carbamates (lifetime days, without or with use in past year)				
0 days	40	45	1.00 (referent)	0 (referent)
1–50 days, no use in past year	29	26	1.42 (1.30–1.56)	1.02 (0.09)
1–50 days, used in past year	5	6	1.18 (1.00–1.40)	0.57 (0.16)
> 50 days, no use in past year	15	14	1.55 (1.38–1.75)	1.22 (0.11)
> 50 days, used in past year	10	10	1.48 (1.27–1.71)	1.15 (0.14)
Pyrethroids (lifetime days, without or with use in past year)				
0 days	72	78	1.00 (referent)	0 (referent)
1–50 days, no use in past year	13	10	1.31 (1.17–1.47)	0.80 (0.12)
1–50 days, used in past year	6	5	1.16 (0.99–1.37)	0.61 (0.16)
> 50 days, no use in past year	4	3	1.24 (1.09–1.58)	0.86 (0.19)
> 50 days, used in past year	5	3	1.31 (1.09–1.58)	1.09 (0.19)

a Proportion of applicators who experienced ≥10 versus < 10 specific symptoms in the year before enrollment. Results are expressed as odds ratio (OR) with 95% confidence interval (95% CI) from logistic regressions adjusted for age, state, education, cigarette smoking, and alcohol use.

b Number of specific symptoms experienced at least once in the year before enrollment, used as a continuous variable. Results are expressed as estimates (SE) from linear regressions adjusted for age, state, education, cigarette smoking, and alcohol use.

c A high-pesticide-exposure event involving internal exposure was defined as one involving inhalation or ingestion of pesticide.

**Table 4:** Associations of specific neurologic symptoms with pesticide exposure among licensed pesticide applicators enrolled in the AHS 1993–1997 (*n* = 18,782).

	Any pesticide	All insecticides	Organophosphates	Organochlorines	Fumigants
Headache	1.25*	1.80*	1.62*	1.31*	1.10
Fatigue	0.99	2.29*	2.34*	1.60*	1.10
Tension	1.22*	1.98*	1.82*	1.55*	1.30*
Insomnia	1.24*	1.78*	1.70*	1.56*	1.21
Irritability	1.16*	2.33*	1.93*	1.57*	1.46*
Dizziness	1.26*	2.34*	1.77*	1.66*	1.38*
Numbness in hands or feet	1.18*	2.64*	2.67*	1.75*	1.25*
Depression	1.19*	2.30*	2.09*	1.68*	1.31*
Nausea	1.11	1.93*	1.88*	1.59*	1.37
Absentmindedness	1.12	2.57*	2.13*	1.75*	1.24*
Difficulty concentrating	1.15*	2.27*	2.07*	1.84*	1.36*
Loss of appetite	1.14	1.80*	1.76*	1.51*	1.22
Excessive sweating	1.12	2.03*	1.84*	1.70*	1.10
Twitches in arms or legs	1.16*	2.65*	2.34*	1.68*	1.43*
Fast heart rate	1.22*	2.19*	1.95*	1.74*	1.23
Weakness in arms or legs	1.01	1.84*	1.82*	1.77*	1.03
Poor balance	1.20*	2.46*	1.77*	1.95*	1.40*
Poor night vision	1.24*	2.06*	1.85*	1.62*	1.38*
Tremor in hands	1.26*	2.20*	2.00*	1.68*	1.17
Blurred/double vision	1.25*	2.08*	1.87*	1.75*	1.29*
Changes in smell or taste	1.61*	2.11*	1.83*	2.12*	1.57*
Difficulty speaking	1.30*	2.25*	1.94*	1.97*	1.07
Loss of consciousness	1.49*	1.41	1.26	1.76*	1.34

Table entries are odds ratios for experiencing the symptom with high frequency compared with low frequency, using cut points shown in Table 2. Odds ratios were calculated by logistic regression models with adjustment for age, state, education, cigarette smoking, and alcohol use. Estimates are for the highest category of lifetime days of use of the indicated pesticide groups.

*Estimates for which the 95% confidence interval excluded 1.00.
